# Jarosite and Alunite in Ancient Terrestrial Sedimentary Rocks: Reinterpreting Martian Depositional and Diagenetic Environmental Conditions

**DOI:** 10.3390/life8030032

**Published:** 2018-08-03

**Authors:** Sally L. Potter-McIntyre, Thomas M. McCollom

**Affiliations:** 1 Department of Geology, Southern Illinois University, Carbondale, IL 62901, USA; 2 Laboratory for Atmospheric and Space Physics, University of Colorado, Boulder, CO 80309, USA; tom.mccollom@lasp.colorado.edu

**Keywords:** jarosite, alunite, Mars, diagenesis, Mollies Nipple, Jurassic, jarosite stability

## Abstract

Members of the alunite group are precipitated at low pH (<1 to ~4) in oxidizing environments, are unstable in circumneutral conditions, and are widespread on Mars. At Mollies Nipple in Kane County, Utah, USA, jarosite and alunite are abundant as diagenetic cements in Jurassic sandstones. This research characterizes the jarosite and alunite cements with the goal of determining their origin, and tests the hypothesis that jarosite and alunite may be more stable than the current understanding indicates is possible. Previous studies have placed the jarosite- and alunite-bearing caprock at Mollies Nipple in the Navajo Sandstone, but the presence of water-lain deposits, volcanic ash, volcanic clasts, and peloids show that it is one of the overlying Middle Jurassic units that records sea level transgressions and regressions. A paragenetic timing, established from petrographic methods, shows that much of the cement was precipitated early in a marginal marine to coastal dune depositional environment with a fluctuating groundwater table that drove ferrolysis and evolved the groundwater to a low pH. Microbial interaction was likely a large contributor to the evolution of this acidity. Jarosite and alunite are clearly more stable in natural environments than is predicted by laboratory experiments, and therefore, the Martian environments that have been interpreted as largely acidic and/or dry over geologic time may have been more habitable than previously thought.

## 1. Introduction and Background

Examination of the Martian surface mineralogy over the last couple of decades has revealed the widespread occurrence of sulfate minerals (including abundant minerals from the alunite group) in a variety of geologic settings [1,2,3,4]. Minerals from the alunite group first came to prominence with the identification of diagenetic jarosite cement within the sedimentary rocks at Meridiani Planum using the Mössbauer spectrometer onboard the Opportunity rover [5,6,7]. However, jarosite and alunite have been identified using spaceborne spectroscopy at a number of locations around the planet [8,9,10,11,12,13,14,15]. In several cases, these minerals are found in layered deposits indicative of deposition by sedimentary processes.

Members of the alunite mineral group are of particular interest for studies of Mars because they have the potential to place definitive constraints on aqueous conditions. For example, members of this group are precipitated at low pH (<1 to ~4) and in oxidizing environments, as described in [16,17]. Additionally, laboratory experiments show that jarosite and alunite are stable only in acidic (pH < 5) fluids and that in the presence of circumneutral fluid, they dissolve rapidly (in days or weeks) e.g., [17,18]. Therefore, the presence of these minerals constrains formation conditions as well as subsequent fluid/rock interactions and leads to interpretations that sites where these minerals are observed on Mars were acidic and remained largely acidic (or dry) for the duration of fluid/rock interactions over geologic time [6,17,18,19,20].

Owing to the potential usefulness of jarosite and alunite as indicators of past aqueous conditions on Mars, considerable study of these minerals in terrestrial analog sites has been conducted in recent years; for example, in [21,22,23,24,25,26,27,28,29,30,31] and many others. On Earth, large deposits of alunite and jarosite are most commonly found in volcanic acid-sulfate systems—where they usually precipitate by alteration of feldspar and other silicates—or in acid mine drainages such as Rio Tinto, where they precipitate by oxidation of pyrite and other metal sulfide minerals in massive sulfide deposits [20,31,32]. While such environments provide the proper geochemical conditions for jarosite and alunite precipitation, most of the layered deposits where these minerals are observed on Mars have no clear association with volcanic processes or sulfide deposits.

Jarosite and alunite can occur in sedimentary rocks, but typically they are only present as minor phases mixed in with an assortment of other minerals. For example, jarosite commonly occurs as a tertiary weathering product of pyrite in sedimentary rocks and is present in minor amounts co-occurring with pyrite and hematite [33,34]. Therefore, it is uncertain whether these environments could account for the jarosite- and alunite-rich deposits that would be required to be detectable on Mars.

**Figure 1:**
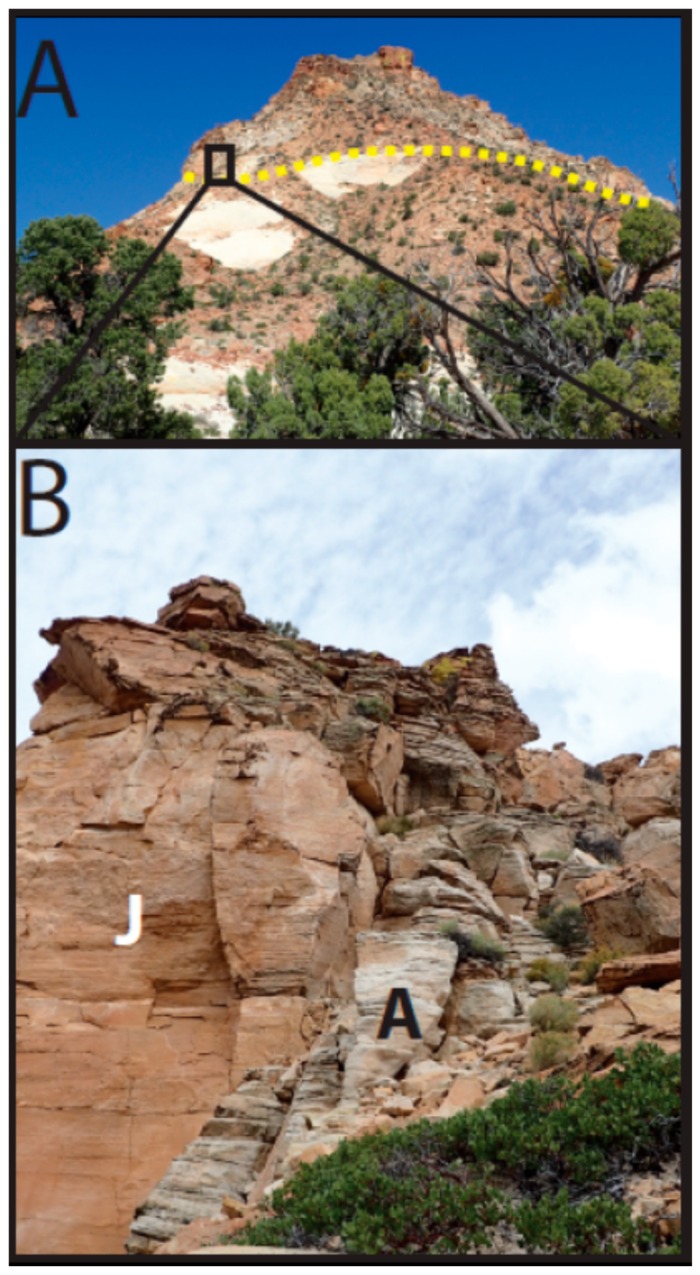
Mollies Nipple study area. (**A**) View of Mollies Nipple from the west looking east. Yellow dotted line separates the caprock from the underlying Navajo Sandstone. Note the float covering the slopes from the erosion of the jarosite-bearing caprock. (**B**) Jarosite (J) and alunite + kaolinite (A) diagenetic facies. The diagenetic facies are segregated by chemistry rather than by any lithological or structural controls.

**Figure 2:**
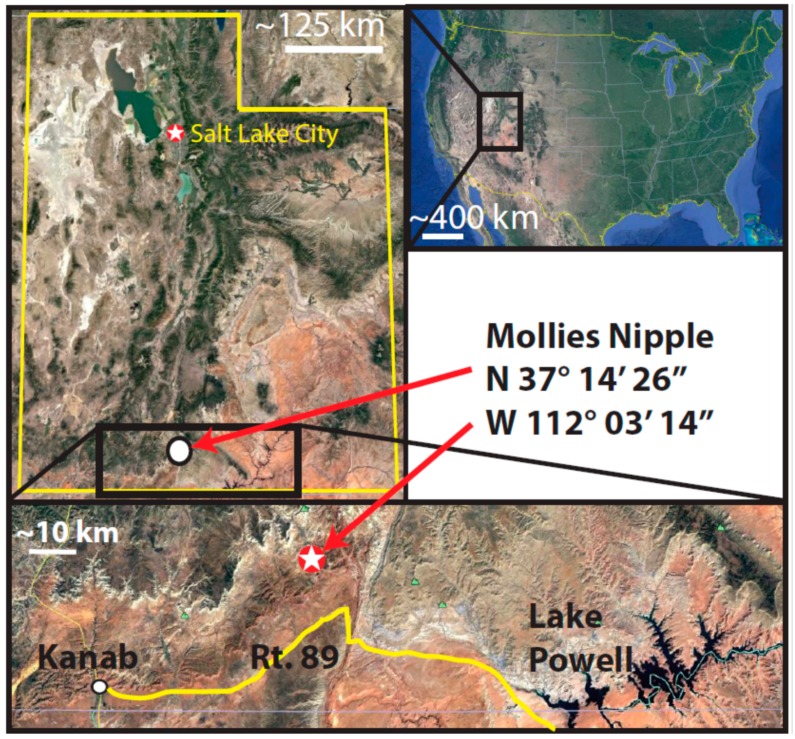
Mollies Nipple location maps at different scales. Upper right shows the U.S. within North America. Upper left shows an inset of the state of Utah with Mollies Nipple located near the southern border (white dot). Lower image is an inset of southern Utah showing Mollies Nipple (white star) in relation to local landmarks, Kanab, Lake Powell, and Route 89 (yellow line).

At Mollies Nipple in Kane County, Utah, jarosite and alunite are abundant as diagenetic cements in Jurassic sandstones (Figure 1 and Figure 2, [28,35]). The butte exhibits strong spectral signatures for jarosite and alunite that are similar to those observed on Mars [28,35]. However, the origin of the jarosite- and alunite-bearing cements at Mollies Nipple is not yet understood. This research investigates the diagenetic history of the cements and their preservation in the geologic record. The long-term persistence of jarosite and alunite at Mollies Nipple suggests that these minerals have likely been exposed to aqueous conditions for much longer periods than the current understanding of jarosite and alunite stability indicates is possible. This research characterizes the jarosite and alunite cements with the goal of determining their origin, and tests the hypothesis that jarosite and alunite may be more stable in circumneutral conditions than previously thought.

It is noted that minerals in the alunite group have the ideal molecular formula AB_3_(SO_4_)_2_(OH)_6_, where the A site is most commonly occupied by the monovalent ions K^+^, Na^+^, and H_3_O^+^ (hydronium) and the B site is primarily occupied by Al^+++^ (alunite subgroup) or Fe^+++^ (jarosite subgroup). In the planetary literature, the terms “jarosite” and “alunite” are widely used to refer to any member of these subgroups, even though these terms formally refer only to the K-bearing endmembers. At Mollies Nipple, the A sites of the minerals are occupied predominantly by K, so the use of jarosite and alunite refers to the specific mineral names.

## 2. Geologic Setting

Mollies Nipple is a prominent butte located in southern Utah that rises ~200 m above the surrounding landscape (Figure 1 and Figure 2). The base of the butte is composed of eolian Jurassic Navajo Sandstone, a fine-grained quartz arenite. At this locality, the early diagenetic hematite grain coatings of the quartz sand grains were mobilized during infiltration by a chemically reducing fluid, resulting in white (“bleached”), friable sandstone presenting as white exposed areas in the lower slopes of Figure 1 [36,37,38,39]. The butte is resistant to erosion owing to the presence of a well-cemented caprock [28].

Spectral analysis of the rocks in the visible to near-infrared (VNIR) region of the electromagnetic spectrum shows prominent adsorption bands for either (1) jarosite or (2) alunite plus kaolinite [28,35]. These spectral signatures are also present in airborne HyMap data, which facilitated the initial discovery of the site and have since allowed the distribution of sulfate minerals to be mapped [28,35]. Although jarosite and alunite are present only in the caprock, float rock eroded from this caprock gives spectral signatures for these minerals over a broad area that includes the lower slopes of the butte. The spectral signatures for jarosite and alunite + kaolinite in these rocks closely resemble those observed by the satellite remote sensing of martian outcrops [28,35].

**Figure 3:**
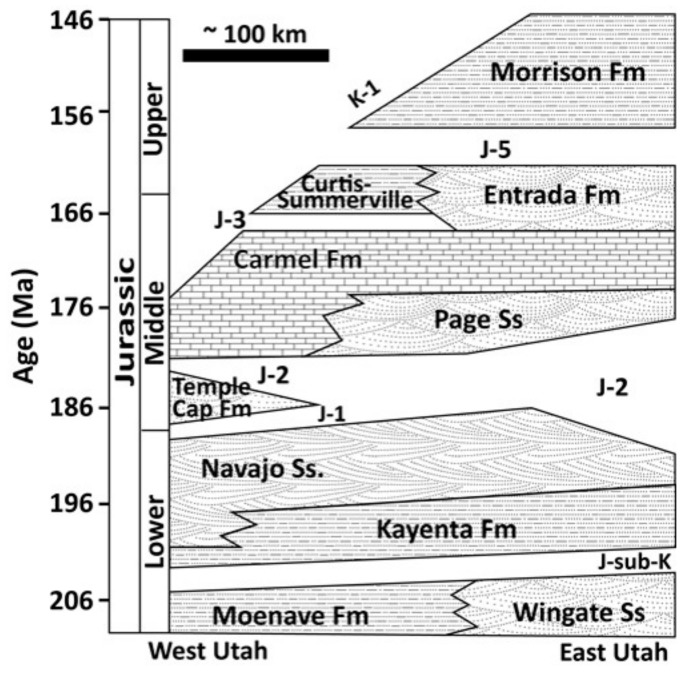
Stratigraphy of southern Utah modified from [39]. The regional unconformities are labelled J-1, J-2, and so on, where J is Jurassic and K is Cretaceous (except for J-sub-K, which means the Jurassic unconformity below the Kayenta Formation). Fm means formation, and Ss means sandstone.

The stratigraphic relations of the cemented caprock to the underlying Navajo Sandstone are uncertain. In previous work, the caprock was interpreted as Navajo Sandstone (via photogeologic mapping), although it lies at an elevation higher than the mapped upper extent of the Navajo Sandstone in the region [35,40]. Throughout the region, the Navajo Sandstone is unconformably overlain by the Carmel and Temple Cap Formations (to the northwest) and the Page Sandstone (to the southeast; Figure 3). The Carmel Formation was deposited in a coastal sabhka and contains thick gypsum beds (<24 m), as well as fluvial, eolian, and tidal facies [41]. The Page Sandstone was deposited adjacent to the Carmel Formation as the marginal marine facies of the Carmel Formation graded into a terrestrial eolian environment [42,43,44]. In the region surrounding the study area, these two units intertongue due to sea level transgressions and regressions, making differentiation sometimes difficult. The Temple Cap Formation is interpreted as a unit where these sabkha and eolian facies are interbedded, but it stratigraphically underlies the Page and Carmel Formations and lies between the J-1 and J-2 unconformities (Figure 3; [45]). The J-2 regional unconformity is present where the Temple Cap Formation is eroded and is marked by a distinctive pebble lag [42]. Where the Temple Cap Formation is not present, either the Carmel Formation or the Page Sandstone overlies the Navajo Sandstone [43,46,47]. However, because the overlying strata have been eroded in the region immediately surrounding Mollies Nipple, and the Page, Carmel, and Temple Cap Formations are all present in the surrounding region, the stratigraphic relations at the study site are uncertain.

The timing and origin of the jarosite- and alunite-bearing cements are also uncertain. Bell and Bowen [28] proposed that the cements were deposited by sulfate-rich hydrothermal solutions, emplaced by fluids migrating along fractures well after the original sandstones had been deposited. Evidence cited in support of this proposition includes mineralized joints that feed lithologically controlled mineralization present in the sandstone around the base of Mollies Nipple (within the Navajo Sandstone), and petrographic evidence suggesting that the cements were precipitated after the sand grains had undergone compaction. However, the mineralized joints occur several tens of meters in the stratigraphy below the jarosite/alunite-bearing caprock, and there is no obvious channel connecting the joints to the overlying caprock (although these could have been present in areas that are now eroded or not exposed). Perhaps more significantly, the mineralization in the underlying Navajo Sandstone is composed of iron oxides/oxyhydroxides, with no jarosite or other sulfates present that would suggest a clear diagenetic relationship with the caprock cements.

## 3. Purpose of Study

The principal goals of this research are twofold: (1) to interpret the origin and paragenesis of the jarosite and alunite cements, and (2) to interpret the depositional environment of the jarosite- and alunite-bearing caprock in order to correctly identify the formation and, therefore, better understand the origin of the diagenetic cement. Understanding the paragenetic relations within the cements will dovetail with the sedimentologic research to illumine the history of the unit. This research seeks to determine the origin of these unusual cements and understand why they have persisted over geologic time in spite of exposure to circumneutral fluids for tens of millions, if not hundreds of millions, of years. This understanding is crucial in order to correctly interpret similar Martian environments and better constrain conditions related to habitability at these sites.

## 4. Methods

Field studies included a characterization of the lithologies above and below the contact zone between the Navajo Sandstone and the overlying, jarosite-bearing unit in order to determine the stratigraphy and document the relations between the diagenetic cement facies. Representative samples were collected and returned to the laboratory for further analyses.

X-ray diffraction (XRD) was performed on both whole rock samples (*n* = 2) and on separates of the fine-grained fraction (*n* = 14) to focus on cements. The majority of the samples (*n* = 12) were performed with a Terra instrument (Olympus, Inc., Tokyo, Japan) using Cu K_α_ radiation at a 0.02°2Θ step from 2–55°2Θ. Analyses were performed on fine-grained fractions separated from the bulk rock by lightly pulverizing the rocks, sonicating the resulting powders in ethanol, and then removing and drying the suspended fraction. In the lab of Potter-McIntyre, samples (*n* = 2) were prepared by disaggregation in a mortar and pestle, placed in a random powder mount, and analyzed with a Rigaku Ultima IV X-ray diffractometer using Cu K_α_ radiation at a 0.02°2Θ step from 2–35°2Θ. Samples were then peptized in a slurry of deionized water (50 mL) and sodium triphosphate (5 mL) to disperse the clays. Samples were centrifuged to separate the <2 µm fraction, and the slurry was air-dried on slides to orient the clays and then analyzed in the diffractometer. The air-dried slides were then put in a desiccator with ethylene glycol for 24 h and reanalyzed. Microprobe analysis was performed at the University of Colorado Boulder to obtain additional chemistry data on the sample, UT16-MN-Jp1, because some X-ray amorphous phases were present.

Representative samples were selected (*n* = 11), and polished thin sections were prepped at Spectrum Petrographics in Washington. Thin sections were analyzed using a petrographic microscope, and paragenetic relations were evaluated. The morphology and spatial relationships of minerals were further characterized using a JEOL 6480LV scanning electron microscope (SEM) operated in back-scatter electron mode. The instrument was equipped with an electron-dispersive X-ray spectrometer (EDS) for semiquantitative determination of chemical composition. The SEM/EDS analyses were performed both on rock fragments mounted on aluminum stubs using double-sided carbon tape and on polished thin sections. Samples that were identified as having jarosite or alunite content (either via XRD or petrography) were selected for EDS. Morphology of the cements and their spatial relationships with the matrix were characterized. The SEM analysis also helped identify the presence of minerals that were present in abundances that were too low to be detected by XRD.

Sample UT16-MN-Jp1 was also examined by electron microprobe analysis (EMPA) to determine the chemical composition of mineral phases. Measurements were performed on a JEOL JXA-8230 instrument in the Department of Geological Sciences at the University of Colorado. Samples were analyzed with an accelerating voltage of 15 kV using a 1 µm beam and a beam current of 10 nA.

## 5. Results

### 5.1. Field Study

**Figure 4:**
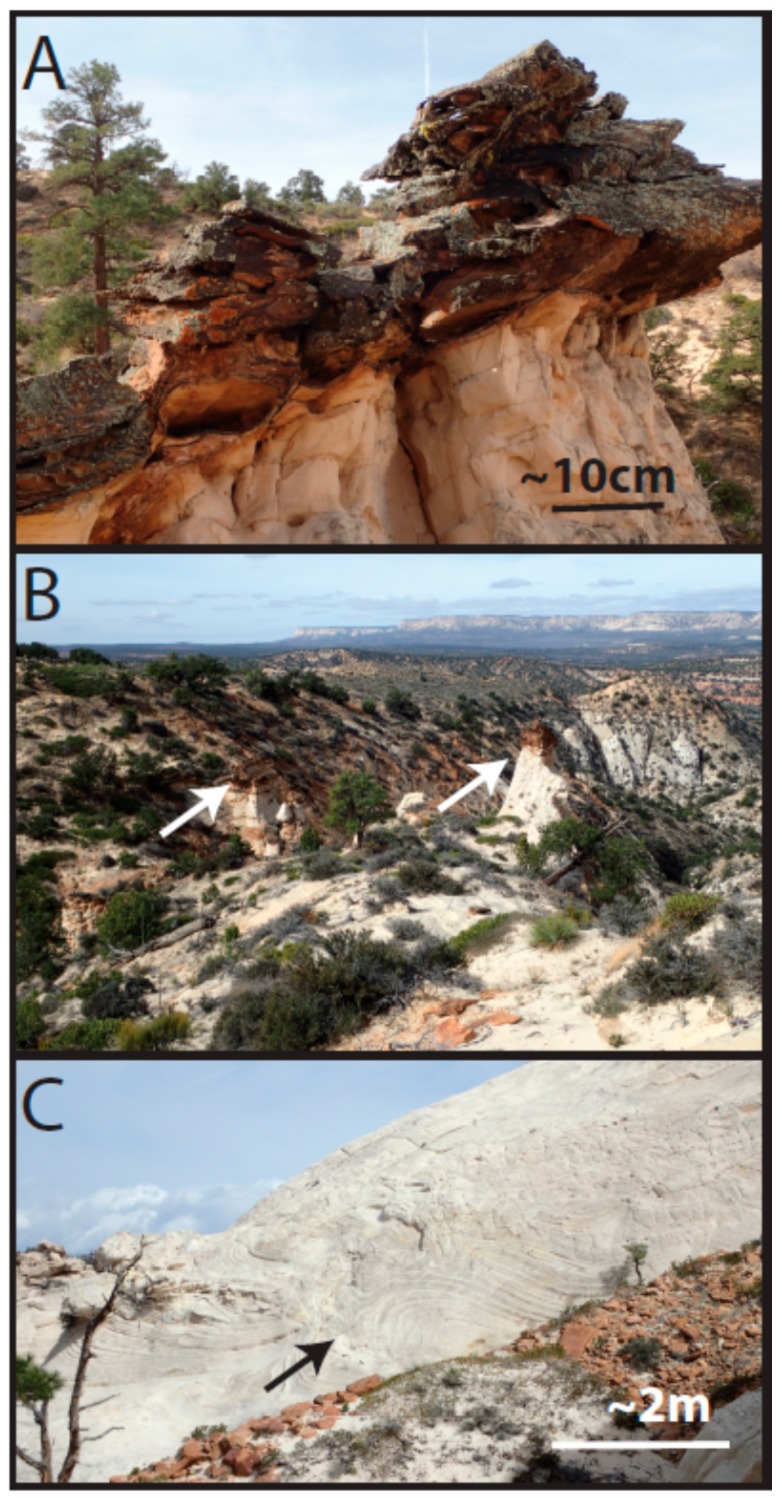
Navajo Sandstone. (**A**) Concretionary iron (oxyhydr) oxide mineralization present in the approximate middle of the unit. (**B**) View looking north from Mollies Nipple with arrows showing location of the mineralization in A. (**C**) Arrow points to large-scale soft sediment deformation characteristic of the upper part of the Navajo Sandstone (that is not present in the overlying caprock).

The basal unit below the caprock at Mollies Nipple is Navajo Sandstone (Figure 1, Figure 2 and Figure 3; [28,35]). The sandstone is a fine-grained, well-sorted, rounded, quartz arenite [48,49,50]. The formation exhibits high-angle cross-stratification with bed sets ~6–15 m thick. Some large-scale (<4 or 5 m thick) soft sediment deformation is present in the bottom half of the section up to about halfway to the caprock, and soft sediment deformation is lacking in the top half of the section. The unit is white with minimal iron (oxyhydr) oxide cement. Halfway between the base of the butte and the contact with the caprock (within the soft sediment deformation facies), iron (oxyhydr) oxide concretionary cement is present in some locations, and this cement is confined to subhorizontal lenses (Figure 4). Some lenses have evenly dispersed cement throughout; however, most exhibit ~1 cm thick heavily cemented layers. Some of the concretionary patterns are “boxworks” that range from ~5 × 10 cm to ~15 × 30 cm. Other mineralization patterns are layers resembling onion skin and are up to 1 × 3 m (Figure 4).

**Figure 5:**
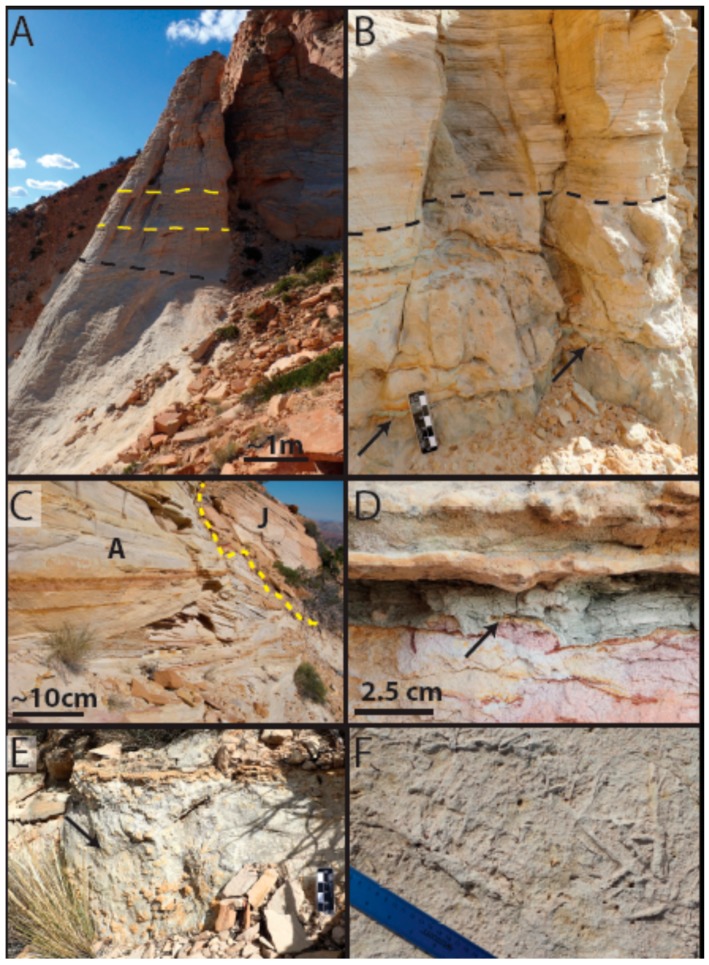
Lithology of the jarosite/alunite-bearing cap unit. (**A**) Black dashed line indicates contact between the Navajo Sandstone and the lower subunit, and the yellow-dashed lines denote bed sets of a much-reduced size compared to the Navajo Sandstone bed sets. (**B**) Highly bioturbated lithofacies with ash lenses (black arrows) overlain by planar horizontally stratified lithofacies (separated by black dotted line; scale bar is 2 cm = 1 mark). (**C**) Eolian lithofacies within the alunite diagenetic facies (**A**) with an outcrop of jarosite diagenetic facies (J) in the background (delineated by dashed yellow line). (**D**) Ash lens (black arrow). (**E**) Highly bioturbated lithofacies within the alunite + kaolinite diagenetic facies (scale bar is 2 cm = 1 mark). (**F**) Close-up showing bioturbation.

**Figure 6:**
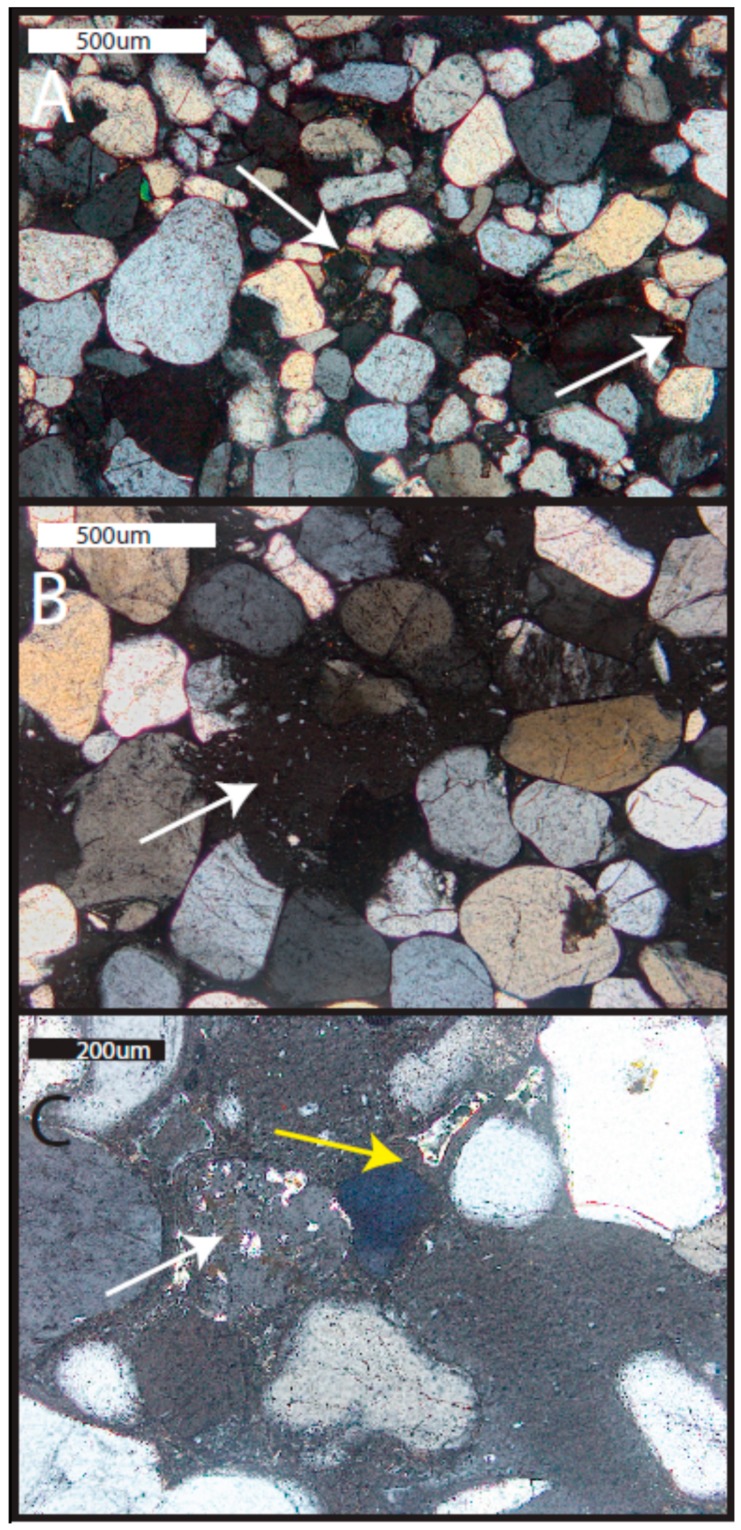
Photomicrographs of Navajo Sandstone and lower subunit of the cap rock (polarized light). (**A**) Navajo Sandstone with iron (oxyhydr) oxide cement and illite rims (white arrows; sample UT15-MN-Jn11). (**B**) Lower subunit of caprock with degraded volcanic clast indicated by porphyritic texture (sample UT16-MN-Jp1). Note the difference in grain size from the Navajo Sandstone example. Volcanic clasts are abundant in both the lower and upper subunits; however, they are lacking in the Navajo Sandstone. (**C**) Volcanic clast (white arrow) and laminated zeolitic mineral with bright illite within the pore space (yellow arrow) in the lower subunit (sample UT16-MN-Jp1).

The caprock unit contains two subunits and is distinguished from the underlying Navajo Sandstone by a supersurface that truncates the underlying bedsets and a subtle reduction in bedset thickness (Figure 5). The lower subunit is ~15 m thick and contains small-scale crossbed sets (~3–4 m thick; Figure 5a). This unit is a moderately well-sorted, sub-rounded, quartz arenite (Figure 6). The contact between the lower and upper subunits is conformable into intercalated planar horizontal laminated siltstones alternating with 1–3 m thick cross-stratified sandstones (Figure 5). The upper subunit is ~25 m thick. Some of the horizontal beds are highly bioturbated. Lenses of clay-rich, volcanic ash (1–2 cm thick by 1–2 m wide) are common. Reworked volcanic ash is identified sedimentologically by thin, laterally restricted, recessively weathered clay within a coarser-grained, water-lain unit (Figure 5D). The sandstones in this unit are fine-grained, well-sorted, and subangular to rounded with a slightly greater number of colored cherts than the Navajo Sandstone. The total caprock is ~40 m thick at its thickest point.

Cementation in caprock is very distinctive, and the upper subunit is well-indurated with predominately jarosite (yellow) cement. Some areas of alunite (white) and kaolinite cement are present that are ~1–4 m wide within the upper subunit, although most of the lower subunit is likely the alunite/kaolinite diagenetic facies. The jarosite and alunite + kaolinite are segregated within the upper subunit; however, some of the selvages contain both jarosite and alunite (see next Section; see Supplementary Materials for XRD results). In some places, jarosite cement is preferentially preserved within wind ripple laminae.

### 5.2. Mineralogy and Petrography

#### 5.2.1. Navajo Sandstone

This unit is a fine-grained, quartz arenite (Figure 6a). Minor feldspar is present (~2%). Grains are rounded to subangular. A minor amount of iron cement is present rimming grains and in pore throats. Some illite rims are present (identified by thin, high birefringence grain coatings). Suture contacts are common.

#### 5.2.2. Lower Subunit of Caprock

**Figure 7:**
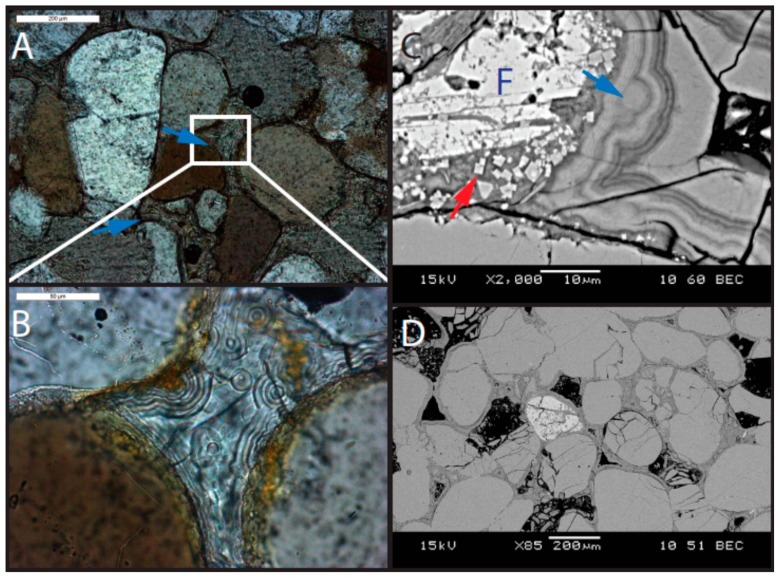
Micrographs of the lower subunit of the cap rock (sample UT16-MN-Jp1). (**A**) Photomicrograph (polarized light) showing laminated second-generation zeolitic mineral (blue arrows). (**B**) Inset of view A (white box in view A) showing laminated, botryoidal cement. (**C**) SEM image with frothy first-generation mineral with embedded euhedral alunite crystals (red arrow) in the secondary pore space created by the dissolution of feldspar grain (F). Infilling the pore space is the laminated, botryoidal zeolitic phase (blue arrow). (**D**) SEM image showing laminated cement completely surrounding the grain.

**Figure 8:**
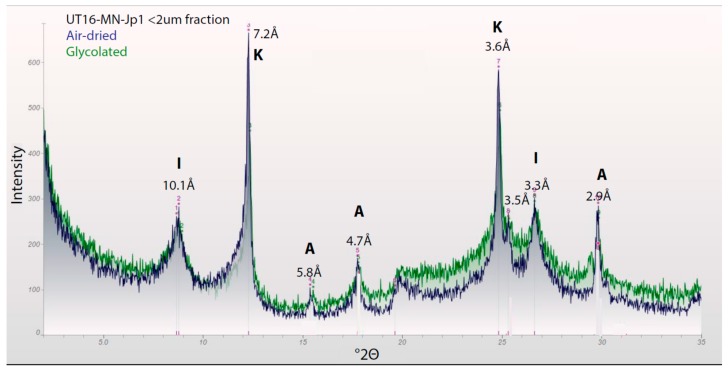
XRD results of the <2 µm fraction of the lower subunit with air-dried and glycolated results. Illite (I) is indicated (rather than interstratified illite/smectite) because the illite peak does not shift after glycolation. Also present are alunite (A) and kaolinite (K). A possible woodhouseite peak is present as a shoulder at 3.5 Å.

**Figure 9:**
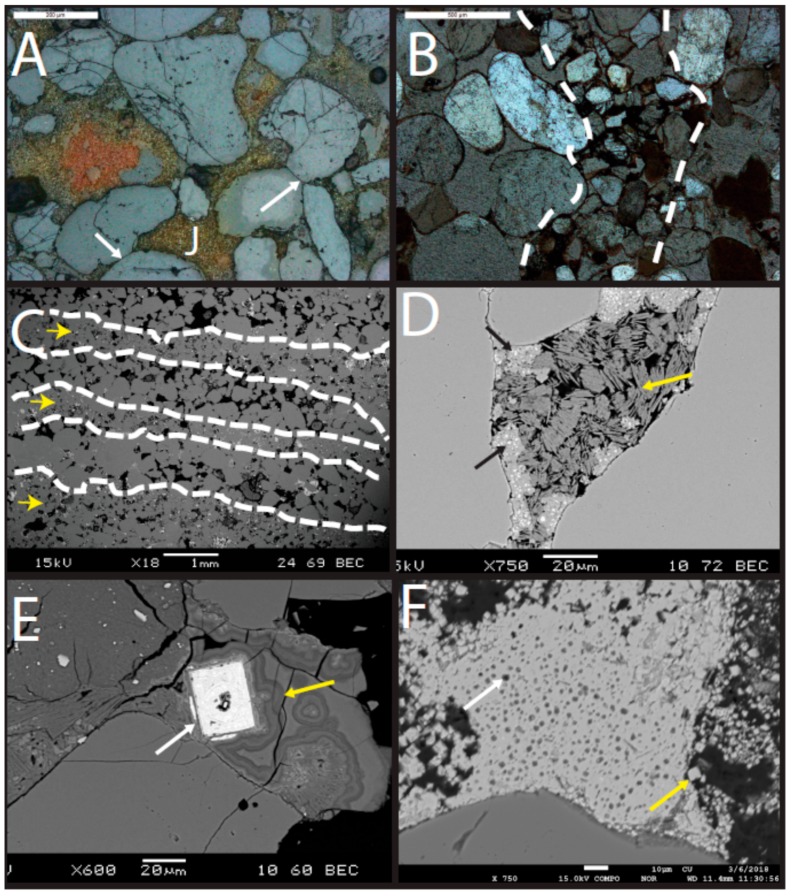
Early diagenetic features. (**A**) Pore space completely filled with jarosite (J) (plane light). Some suture contacts are present between quartz grains (blue arrows). Reddish cement is jarosite degrading to iron (oxyhydr) oxide (sample UT16-MN-Jp4). (**B**) Plane light; sample UT16-MN-Jp7 and (**C**) SEM; sample MN5ts. Jarosite preferentially preserved within finer-grained wind ripple laminae (white dotted lines and yellow arrows). (**D**) Alunite + kaolinite diagenetic facies. The alunite in this sample have jarosite cores, which appear bright in this back-scattered SEM image (black arrows). Yellow arrow shows kaolinite (sample UT16-MN-Jp2). (**E**) Large euhedral cubic crystal that is possibly a siderite or hematite after pyrite pseudomorphs (white arrow) in the lower subunit. Large euhedral crystals are commonly precompaction features, but this crystal is also surrounded by the laminated second-generation mineral (yellow arrow), providing further evidence for an early origin (sample UT16-MN-Jp1). (**F**) Jarosite crystals (yellow arrow) with dark alunite cores (white arrow) in back-scattered electron image (sample MN10).

This unit is a fine- to medium-grained quartz arenite, slightly coarser than the underlying Navajo Sandstone (Figure 6). Minor feldspar is present (~1%), but it is less common than in the Navajo Sandstone. Volcanic clasts are present (identified by phenocrysts within a groundmass) and some are degraded such that the groundmass is absent (Figure 6). Grains are well-rounded, but commonly exhibit dissolution (including quartz grains; Figure 7). Some suture contacts are observed; however, most grains are rimmed with a laminated isopachous cement with little to no contact between grains (Figure 6). The isopachous cement is 10–15 µm thick and has botryoids in places (Figure 7). Quartz grains and even feldspars in the lower subunit do not exhibit the degree of dissolution observed in the overlying strata. However, feldspars are typically partially dissolved and the secondary porosity created by dissolution is filled with illite, alunite (with some minor woodhouseite), and kaolinite (Figure 7 and Figure 8). Several large crystals (~30 µm on the long axis) with cubic or pseudocubic habits are present in this basal section that could either be primary siderite or, more likely, pseudomorphs of either hematite or siderite after pyrite (Figure 9E).

Viewed with higher resolution microscopy (SEM), the cement in the lower subunit exhibits three generations of precipitation: (1) a first-generation frothy illite with euhedral alunite and woodhouseite plus kaolinite crystals embedded in the illite; (2) a second-generation isopachous, laminated, Ca-rich phase that is likely an amorphous aluminosilicate (possibly zeolite) containing 2–4 wt % As_2_O_3_; and (3) a third-generation infilling of porosity by illite (Figure 7, 8D; see Supplementary Materials for XRD and geochemistry). The second generation laminated phase has the appearance of zeolite, but it has a composition closer to smectite with an average molar (Ca + K + Na): (Si + Al) ratio of around 0.04, rather than the higher ratios expected for zeolites (0.11–0.17) (see EMPA results in Supplementary Materials). However, no smectite or interlayered illite/smectite is observed with XRD (Figure 8); therefore this phase is likely an X-ray amorphous, zeolitic, Ca-rich aluminosilicate.

#### 5.2.3. Upper Subunit of Caprock

**Figure 10:**
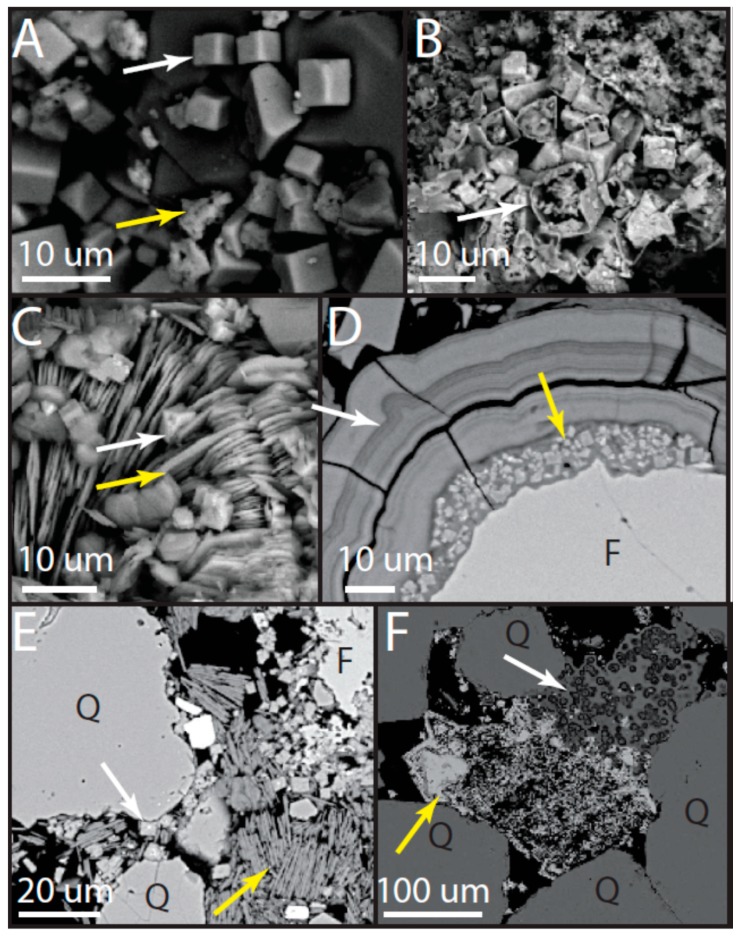
SEM back-scattered electron images of jarosite and alunite cements. (**A**–**C**) are from grain mounts, while (**D**–**F**) are from polished thin sections. (**A**) Upper subunit within the jarosite diagenetic facies showing euhedral, pseudocubic jarosite (white arrow) and amorphous silica blebs (yellow arrow). Dark gray minerals are quartz (sample MN5). (**B**) Iron oxide pseudomorphs after jarosite in the upper subunit (arrow; sample MN5). Remnants of jarosite are present in the interiors of some of these structures. (**C**) Euhedral, pseudocubic alunite (white arrow) on kaolinite (yellow arrow) within alunite + kaolinite diagenetic facies in the upper subunit (sample MN7). (**D**) Potassium feldspar grain (F) in the lower subunit with the frothy, first-generation mineral with embedded euhedral alunite and woodhouseite crystals (yellow arrow) and surrounded by laminated, second-generation mineral (white arrow; sample UT16-MN-Jp1). (**E**) Alunite + kaolinite diagenetic facies in the upper subunit showing quartz (Q) and feldspar (F) dissolution (sample UT16-MN-Jp2). The white arrow points to alunite crystal and the yellow arrow points to kaolinite. The white spot on the alunite crystal is beam damage from EDS analysis. (**F**) Spheroidal forms in silica that are potentially microbial body fossils (white arrow) adjacent to jarosite with unusual crystal habits (yellow arrow; sample MN5).

The upper subunit contains two lithofacies: cross-stratified sandstone and horizontally laminated siltstone. In the cross-stratified lithofacies, grains are very fine to medium, moderately sorted, and rounded to sub-rounded (Figure 10). Some samples exhibit silt wind ripple lamina ~1 mm thick intercalated with medium sand grain flow lamina (~3 mm thick; Figure 10). The siltstone facies is well-sorted and subangular. The composition of the upper subunit is dominantly quartz, with minor feldspar, volcanic clasts (Figure 6B,C and Figure 7A,C), and rare mica and green, glauconitic peloids (Figure 10). Chert (cryptocrystalline quartz) is common.

*Jarosite diagenetic facies*: Where jarosite is present, the pore space can be completely filled with cement; however, where intercalated wind ripple and grain flow laminae are present, cement is commonly present only in the wind ripple laminae (Figure 10D,E). Some small spots of oxidized iron (presumably hematite) are present where jarosite is degrading (Figure 9A). These oxidized spots range from a few µm to >100 µm in diameter. In the alunite facies, cement is similarly abundant where grains are moderately well-sorted or present only in the wind ripple laminae. Illite rims are common in the alunite facies (and observed in XRD patterns). Quartz grains exhibit substantial dissolution in this entire subunit. Some feldspars are nearly or completely dissolved in the alunite facies, leaving “ghost” grains that are replaced with kaolinite and alunite.

Stubs of jarosite diagenetic facies samples contain pseudocubic crystals of jarosite ranging from <1 to >30 µm on the long axis (Figure 10A). Silica blebs (<5 µm diameter) are present (Figure 10A). In some areas, degradation of jarosite crystals is observed, leaving the crystal sides intact, but dissolving the interiors (Figure 10B).

In one thin section sample, a large mass of jarosite is observed near a cluster of spheres within SiO_2_ (Figure 10F). The jarosite exhibits unusual small, oriented laths directly adjacent to the spheres. Some of this jarosite in this sample contains up to 3 wt % As_2_O_3_, although in most crystals it is >0.2 wt %. The spheres are hollow with interior and exterior walls. These features are composed of silicon dioxide (established via EDS). The interior spheres are ~3 µm in diameter (although some are ~2 µm). The spheres are enclosed in a void space between the interior and exterior walls, which are ~2 µm thick, so the entire sphere is ~5 µm in diameter. The smaller spheres are connected and form doublets.

**Figure 11:**
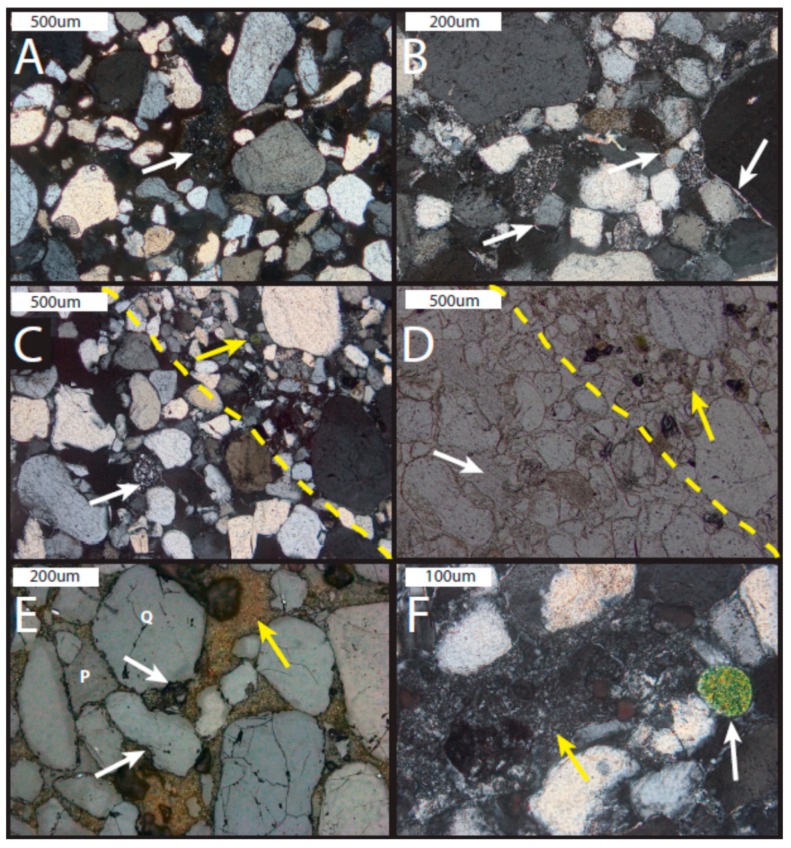
Photomicrographs of the upper subunit of the caprock. (**A**) Upper subunit with volcanic clast (arrow) and jarosite cement (polarized light; samples UT16-MN-Jp4). (**B**) Thin, high birefringence rims around grains are illite (white arrows; sample UT16-MN-Jp5). (**C**) Grain flow lamina (larger grains) with adjacent wind ripple lamina (smaller grains; yellow dotted line separates the two laminae; polarized light; sample UT16-MN-Jp5). (**D**) Plane light image of C showing alunite + kaolinite cement preserved in the wind ripple lamina (yellow arrow) and porosity lacking cement in the grain flow lamina (white arrow; sample UT16-MN-Jp5). (**E**) Jarosite cement (yellow arrows and quartz grains showing dissolution features (white arrows). Note the large pore spaces lacking in cement (labeled P; Q = quartz grain; sample UT16-MN-Jp7). (**F**) Peloid (white arrow) within the alunite + kaolinite (yellow arrow) diagenetic facies (sample UT16-MN-Jp5).

Some jarosite crystals exhibit multiple stages of radial growth. Additionally, although jarosite and alunite are typically segregated, some thin sections of the alunite facies exhibit pore spaces that are completely filled with <10 µm alunite crystals, and many of these crystals have jarosite cores (Figure 11). Interestingly, other examples show jarosite crystals with alunite cores (Figure 9).

*Alunite + kaolinite diagenetic facies*: Stubs of rock samples from the alunite + kaolinite diagenetic facies contain pseudocubic crystals of alunite (<10 µm on the long axis) and platy crystals of kaolinite (<10 µm long) in close association with the alunite (Figure 10C). Fibrous clusters of Fe-rich glauconite are present, but not common. Feldspars exhibit dissolution along cleavage, with kaolinite and alunite precipitated within void space.

Some major differences in mineralogy are apparent between the lower subunit and the upper subunit, even though we classify the lower subunit as the alunite + kaolinite diagenetic facies. The lower subunit has much less alunite + kaolinite than the upper subunit facies, and the lower unit also contains woodhouseite, which has not been observed in any samples from the upper subunit. However, kaolinite is more abundant in the lower subunit (see XRD data in Supplementary Materials).

## 6. Interpretation

### 6.1. Stratigraphy

Previous interpretations placed the entire Mollies Nipple butte, including the caprock, within the Navajo Sandstone [35,40]. However, several features are inconsistent with this interpretation, including the presence of volcanic ash lenses, volcanic clasts, the reduction in bed set thickness, and the presence of peloids. Instead, it is probable that this unit belongs to one of the overlying formations in the region: the Temple Cap Formation or the Carmel Formation, and these two possible interpretations are discussed below (The Page Sandstone is also present in the region; however, this is an entirely eolian unit, so it is not likely the caprock here). The caprock in this area is difficult to interpret because: (1) the rest of the unit is eroded everywhere nearby except for the caprock atop Mollies Nipple, making a thorough stratigraphic characterization difficult, if not impossible; and (2) even where present, the regional stratigraphy is complex in this interval; for example, see [42,43,44,45]. Nevertheless, the peloids that are common in the unit suggest a marine-influenced depositional environment, and the presence of abundant ash and volcanic clasts clearly indicate that it is not the Navajo Sandstone.

The Temple Cap Formation is commonly interpreted as an intertonguing of the eolian Page (dominant in the eastern (landward) part of the basin) and the more tidally-influenced Carmel Formation present in the western (seaward) part of the basin [45,47], and indeed, this is what these units in the caprock at Mollies Nipple seem to be. At the type section ~40 km southeast from Mollies Nipple, the White Throne Member of the Temple Cap exhibits planar horizontal, siltstone beds intercalated with high-angle cross-stratified bed sets that are up to 6 m thick with common volcanic ash lenses [42,45]. Although this lithological description is very similar to the caprock at Mollies Nipple, one problematic issue with this interpretation is that the Sinawava Member of the Temple Cap Formation underlies the White Throne Member and separates it from the Navajo Sandstone. The Sinawava Member forms an obvious red slope between the Navajo Sandstone and the White Throne Member, and this unit is not present at Mollies Nipple [42]. Nevertheless, the presence of peloids suggests an environment adjacent to a marine environment [51], and the upper subunit is likely an intertonguing of more marine facies (i.e., tidal flat) with eolian facies that is characteristic of the Temple Cap Formation [45].

Another possibility is that the unit is the Judd Hollow Member of the Carmel Formation [42,45], although the Judd Hollow Member is sometimes grouped into the Page Sandstone [43]. The Judd Hollow Member contains sandstone and siltstone and represents a transgressive sequence that grades into the eolian Page Sandstone towards the east (landward) [42,45]. A basal limestone is present in the western extent of the unit, but this is absent in the eastern part [45]. Limestone is lacking in the caprock at Mollies Nipple; however, this does not rule out the possibility that this unit is the Judd Hollow Member, because the southern edge of this unit is very sandy owing to an influx of sand from the south [45]. This sand would choke off the carbonate factory and could also account for the siliciclastic, tidal flat facies. Additionally, in Capitol Reef (~100 km to the northeast), the Judd Hollow Member is reported to have ~10 m of eolian sandstone at the base, so this upper subunit overlying a basal eolian sandstone is also consistent with this interpretation [45]. One drawback for this interpretation is that the contact between the Judd Hollow Member and the Navajo Sandstone is characterized by a lag deposit of angular to subangular, coarse to very coarse grains to very fine pebbles—that is, the J-2 unconformity [42]—but this is lacking at Mollies Nipple.

### 6.2. Paragenesis

Paragenetic relations within the caprock show evidence for multiple cement precipitation events throughout the diagenetic history, and some compaction features clearly predate cementation where suture contacts are present. However, evidence presented here supports an early diagenetic interpretation for precipitation of some cements, such as grains completely encased (“floating”) in cement and jarosite preferentially preserved in the wind ripple laminae. We discuss the lower and upper caprock paragenetic histories separately here because of some obvious differences, but there is no lithological boundary that segregates the cements; therefore, the fluids likely evolved at the same time and were interconnected. It is likely that feldspar dissolution throughout the lower and upper subunits provided K and Al for precipitation of illite, alunite, kaolinite, and jarosite, and interestingly, very little feldspar remains in the jarosite facies. All feldspars throughout both subunits exhibit some degree of dissolution.

#### 6.2.1. Lower Subunit of Caprock

Because the division of the cap unit into two subunits was not fully recognized until detailed examination in the lab, the lower subunit was undersampled. However, based on the sample analyzed, three generations of early diagenetic cement precipitation are established within the lower subunit. The first-generation cement is a coprecipitation of frothy illite encasing kaolinite, alunite, and woodhouseite (Figure 7C). Abundant volcanic ash and clasts were being input into the basin throughout this time [51], and these quickly devitrified in water, precipitating zeolite in most Middle to Late Jurassic units in the basin [52,53]. It could be that this ash contained abundant biotite during this depositional time, which altered to illite with some contribution from the alteration of orthoclase clasts, because abundant illite is present (Figure 8), particularly in the lower subunit and in the lower section of the upper subunit (see Supplementary Materials). Although illite is typically stable at more alkaline pH, it is reported in Western Australian acid saline lakes as coprecipitating with jarosite, alunite, and kaolinite [54]. A second-generation, laminated amorphous zeolitic mineral forms isopachous rims around grains and encases the first-generation illite (Figure 7C). Lastly, retained porosity is infilled by a third generation of illite precipitation.

The following paragenetic stages are interpreted for the lower subunit:

1. Precompaction early precipitation of pyrite or siderite. This precompaction likely occurred based on large euhedral crystals that do not exhibit signs of displacive growth (Figure 9).
2. Precipitation of illite, alunite, woodhouseite, and kaolinite.
3. Rising pH (driven, in part, by dissolution of orthoclase, but also the evolution of the fluid discussed below) caused a second phase of precipitation of laminated, second-generation zeolitic mineral.
4. Late-stage infilling of retained porosity by a third-generation event that precipitated illite.

#### 6.2.2. Upper Subunit of Caprock

The upper subunit exhibits features that suggest a complex history of diagenesis beginning early (Figure 9C,D and Figure 11B,C), but continuing through burial diagenesis (Figure 10B,E and Figure 11A,F). Early diagenetic features in the upper subunit include cement preferentially retained in the finer-grained wind ripple laminae and grains “floating” in cement (Figure 5, Figure 8 and Figure 11). The jarosite cement within the wind ripple laminae suggests that cement was precipitated precompaction throughout the unit and then preferentially preserved during subsequent fluid–rock interactions that dissolved cement in the more porous and permeable grain flow laminae. Additionally, cements completely surround grains within the wind ripple laminae, which also suggests a precompaction origin for this cement.

Later-stage burial diagenetic features also are common within the upper subunit. Evidence of long-term exposure to diagenetic fluid–rock interactions include the fact that feldspar is lacking in the jarosite diagenetic facies. Also, quartz grains are degraded in this unit, showing evidence of dissolution (Figure 11E).

One interesting burial diagenetic feature is that some of the alunite crystals have jarosite cores, while some jarosite crystals have alunite cores (Figure 9D,F). This suggests complex fluid events that took place in an evolving environment where acidity and fluid chemistry were fluctuating. Additionally, some jarosite crystals exhibit partial degradation (although they retain a jarosite signature rather than a hematite signature, as would be expected if they were completely oxidized.)

The following paragenetic sequence of events is interpreted for the upper subunit:

1. Jarosite and alunite + kaolinite precipitation throughout the unit. This is interpreted because of precompaction features such as “floating” grains in cement and the preferential preservation of jarosite and alunite that occurs in the wind ripple laminae. Additionally, the presence of cores of jarosite within alunite and vice versa show multistage precipitation events.
2. Preferential removal of jarosite cement within grain flow laminae and further degradation of jarosite during burial diagenesis.
3. Late-stage diagenesis to weathering results in hematite precipitation in spots within the jarosite diagenetic facies.

## 7. Discussion

Diagenetic jarosite and alunite cements at Mollies Nipple present some perplexing problems: (1) How did the early diagenetic fluids become so sufficiently acidic as to precipitate jarosite? and (2) Why are these unstable minerals phases (jarosite and alunite) still present after exposure to circumneutral meteoric water for millions of years? Here, we present a model for the acidification of early diagenetic fluid first, and then propose some possible scenarios for the long-term stabilization of these minerals.

### 7.1. Evolved Groundwater by the Ferrolysis Model

**Figure 12:**
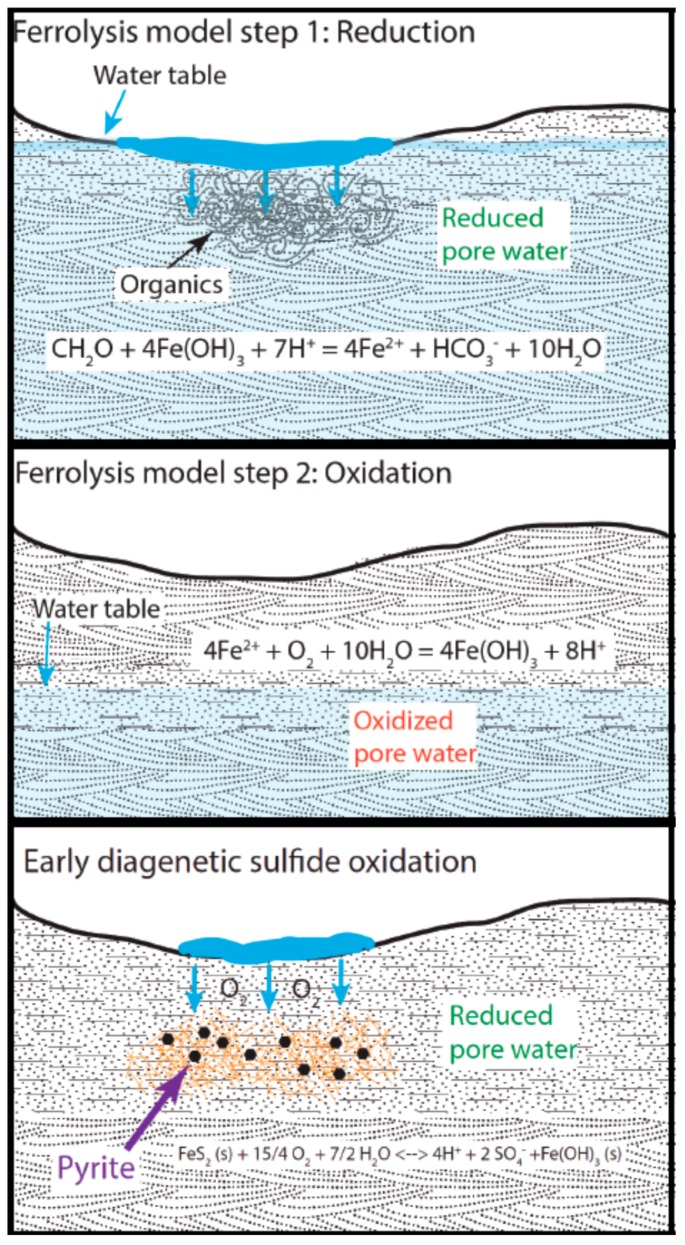
Evolved fluid model showing the two-step process of ferrolysis and early diagenetic sulfide oxidation that possibly also contributed to acidity. The first step occurs when the water table is high and organic matter is present to provide a source for electrons. Then, when the water table drops, the conditions in the shallow subsurface become oxidizing, and this produces more acidity than is consumed in Step 1. Sulfide oxidation may have also contributed some acid to the system. Microbial involvement in these processes would have sped up the reactions to allow the acidity to overcome any buffering by ash or carbonate.

This preliminary model for the development of acidity is based on modern acid saline lake systems in Western Australia (Figure 12) [55,56,57], although the production of sulfuric acid by oxidation of sulfides is considered the driving producer of acidity in those settings [58]. The model for the evolved fluid chemistry at Mollies Nipple cannot call on the same process of oxidation of sulfides as the major source of acidity owing to the lack of sulfide minerals in the sediments. This model does, however, use some of the other sources for acidity, such as ferrolysis as well as microbial sulfate reduction.

The depositional setting at Mollies Nipple in the upper subunit was characterized by regressive and transgressive episodes documented in the lithology as horizontally stratified and bioturbated structures interbedded with eolian facies. The depositional setting was a restricted marine/coastal sabkha environment that was arid and highly evaporative [42,43,45,47,59]. Abundant volcanic ash was input, evidenced by volcanic clasts and ash lenses. This ash would have devitrified and input ions into a high water table that intersected the surface [52,53]. The water would likely have contained some dissolved sulfate in it owing to input from the nearby sea. Iron is the fourth most common element in the Earth’s crust and it is an abundant mineral in these Jurassic rocks, sourced from the early breakdown of ferromagnesian minerals in sediments [38,39].

Ferrolysis would proceed in a two-step process, first reducing, then oxidizing [60]. The first step requires a high water table with organic matter. Organic matter would be expected in this marginal marine depositional environment, and the highly bioturbated facies document the presence of biota. Reduction occurs with organic matter (where “CH_2_O” represents average organic matter) providing the source of electrons in the following equation:

CH_2_O + 4 Fe(OH)_3_ + 7 H^+^ → 4 Fe^2+^ + HCO_3_^−^ + 10 H_2_O (1)

Subsequent sea level regression would cause a drop in the water table, recorded in the rocks as eolian deposition. As the water table drops, the conditions in the shallow subsurface become oxidizing and this produces acidity:

4 Fe^2+^ + O_2_ + 10 H_2_O → 4 Fe(OH)_3_ + 8 H^+^ (2)

Repetition of this process over geologic timescales would produce acidity, as the oxidation reaction produces more acid than is consumed by the reduction. Reaction of the H^+^ with volcanic ash may initially buffer the acidity, but the buffering capacity of the ash will eventually be overcome. It is important to remember that this evolution of groundwater occurred on geologic time scales (over millions of years); however, groundwater recharge rates are measured on human time scales. Therefore, the model for evolved groundwater does not happen in two steps, but rather the repetition of these (human time scale) steps over millions of years as deposition is ongoing.

Of course, it would be expected that dissolved HCO_3_^−^ in a marginal marine environment would buffer the acidity created by the above reactions; however, two possibilities exist to explain the evolution to a highly acidic fluid. One is that the initial fluid composition lacked HCO_3_^−^ either because it precipitated as carbonate minerals early and was eroded, or the carbonate precipitated elsewhere and the more inland fluid was deposited via sea spray that lacked carbonate ions [58]. Indeed, carbonate minerals are common in the Carmel Formation to the west, although they are lacking in the more eastern portions of the unit near Mollies Nipple [45]. The other possibility is that once the pore water fell below a pH of 4.2, the carbonate and bicarbonate ions are all converted to carbonic acid, which further increased acidity in the following steps. This would be very easily accomplished, particularly if there was little carbonate to begin with.

H^+^ + CO_3_^2−^ → HCO_3_^−^ (3)

H^+^ + HCO_3_^−^ → H_2_CO_3_ (4)

Microbial processes may also have been involved in the evolution of this fluid. The rate of sulfide oxidation catalyzed by bacteria is increased by six orders of magnitude relative to their abiotic sulfide oxidation rates [60]. Sulfate reducers commonly occur in conjunction with sulfide oxidizers [61], so it is likely that biological sulfur cycling was a large component in the evolution of acidity. As there was organic matter in the system, this is reaction that could have occurred to produce acidity faster than the abiotic reactions:

SO_4_^2^^−^ + CH_3_COOH → HS^−^ + 2 HCO_3_^−^ + H^+^ (5)

This reaction produces bicarbonate; however, the HS^−^ quickly binds to metals (where Me^2+^ represents metals such as iron that were present in the water under reducing conditions):

HS^−^ + Me^2+^ → MeS + H^+^ (6)

This reaction not only produces protons; it leads to the oxidation of iron sulfide minerals, which further increases acidity. The petrography suggests that very early pyrite precipitation may have happened, so there may have been early pyrite from marine pore water sources as well as the biomediated iron sulfides:

FeS_2_ + 15/4 O_2_ + 7/2 H_2_O → 4 H^+^ + 2 SO_4_^2^^−^ + Fe(OH)_3_ (7)

### 7.2. Jarosite and Alunite Stabilization

The rocks directly overlying the Navajo Sandstone are ~170 million years old, so any cements precipitated during early diagenesis would have been exposed to circulating fluids for this length of time [45,59]. Even if the cements precipitated during burial diagenesis long after the original deposition of the sediments, these minerals have been exposed to at least 10 Ma of circumneutral meteoric water during uplift and exposure on the Colorado Plateau [62]. However, laboratory studies show that jarosite and alunite degrade rapidly (on the order of days or weeks) under circumneutral conditions, and it is inferred that the lifetimes for jarosite grains the size of those observed at Mollies Nipple are less than a few decades [16,17,18,63,64,65]. Because there is no evidence that the jarosite and alunite are tertiary weathering products, the question remains as to why these cements have persisted far beyond the lifetimes suggested by laboratory experiments.

The persistence of jarosite and alunite in the caprocks suggests that previous estimates have underestimated the stability of these minerals in natural settings. It is likely that—similar to other metastable iron minerals such as ferrihydrite—jarosite and alunite can be stabilized by the addition of molecules, trace elements, or organic polymers [66,67,68,69,70]. More work needs to be done to determine precisely what might be stabilizing the jarosite and alunite phases at Mollies Nipple; however, we suggest that the sorption of arsenate is possibly contributing to stabilization. Arsenate can stabilize ferrihydrite [32,66,71], and some bulk samples of the caprock contain more than 2000 ppm As [28]. However, As is below detection levels in many of the jarosite samples collected for this study, so other factors must be involved. Organic polymers can also stabilize ferrihydrite [68], and potential microbial fossils are present in this unit. Additionally, the fluid evolution to such an acidity would likely require microbial involvement, so organic polymer inclusion also remains a candidate for stabilization.

In conclusion, this study shows that jarosite and alunite in the caprocks at Mollies Nipple are preserved over geologic time scales despite being exposed to circumneutral fluids. This finding suggests that previous interpretations of Mars diagenetic environments as being hyperacidic or lacking significant contact with fluids on geologic time scales, based on the presence of jarosite and/or alunite, may be incorrect. These environments may have had much more circumneutral (and therefore more habitable) diagenetic histories than previously imagined. Additionally, this study demonstrates the importance of using stratigraphic and diagenetic data in concert to correctly interpret the details of the depositional and diagenetic history.

## Supplementary Materials

Supplementary Materials are available online at http://www.mdpi.com/2075-1729/8/3/32/s1.
