# Supplementary material for: Jarosite and Alunite in Ancient Terrestrial Sedimentary Rocks: Reinterpreting Martian Depositional and Diagenetic Environmental Conditions
Source: Life (Basel). 2018 Aug 3;8(3):32. doi: 10.3390/life8030032 (PMC6160914; doi:10.3390/life8030032)
Supplement: Supplementary file 1 [file life-08-00032-s001.pdf]

Mar 14 2017 MN UT16-MN-Jp1

14/03/2017 10:47:40

Sample: Jp1 1

Type: Default

ID:

Spectrum processing :

No peaks omitted

Processing option : All elements analyzed (Normalised)

Number of iterations = 5

Standard :

C CaCO3 1-Jun-1999 12:00 AM

O SiO2 1-Jun-1999 12:00 AM

Al Al2O3 1-Jun-1999 12:00 AM

Si SiO2 1-Jun-1999 12:00 AM

S FeS2 1-Jun-1999 12:00 AM

K MAD-10 Feldspar 1-Jun-1999 12:00 AM

Fe Fe 1-Jun-1999 12:00 AM

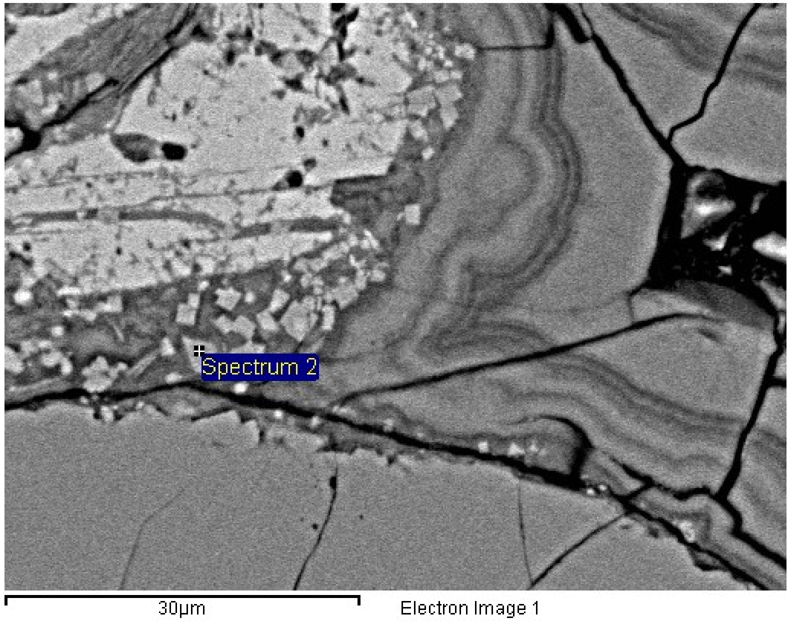

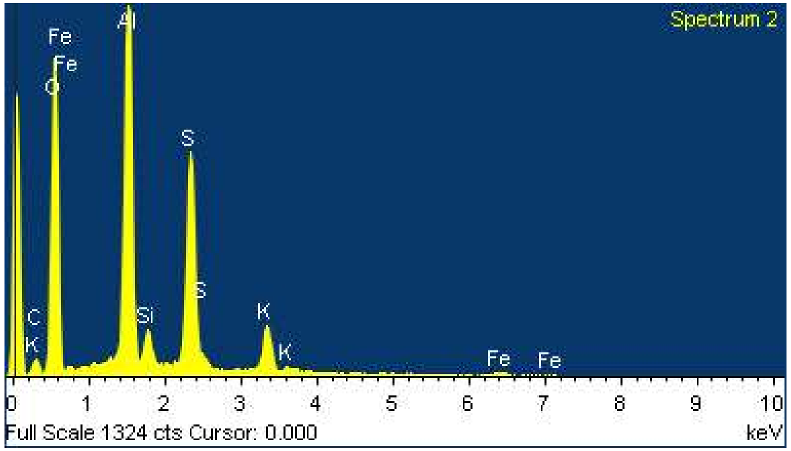

Element	Weight%	Atomic%
C K	8.67	13.87
O K	49.87	59.88
Al K	17.93	12.77
Si K	2.16	1.48
S K	15.59	9.34
K K	4.61	2.26
Fe K	1.17	0.40
Totals	100.00	

Mar 14 2017 MN UT16-MN-Jp1

14/03/2017 10:48:35

Sample: Jp1 1

Type: Default

ID:

Spectrum processing :

No peaks omitted

Processing option : All elements analyzed (Normalised)

Number of iterations = 4

Standard :

C CaCO3 1-Jun-1999 12:00 AM

O SiO2 1-Jun-1999 12:00 AM

Na Albite 1-Jun-1999 12:00 AM

Al Al2O3 1-Jun-1999 12:00 AM

Si SiO2 1-Jun-1999 12:00 AM

K MAD-10 Feldspar 1-Jun-1999 12:00 AM

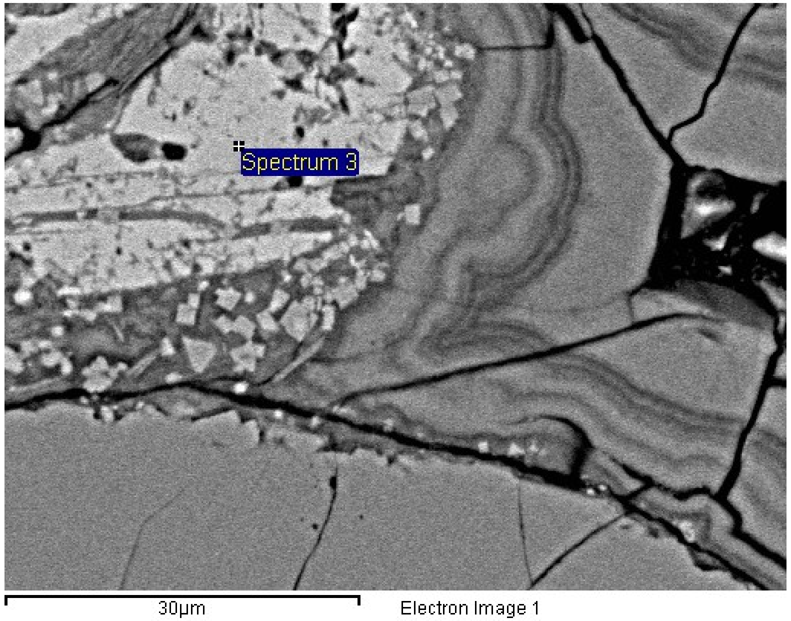

Electron Image 1

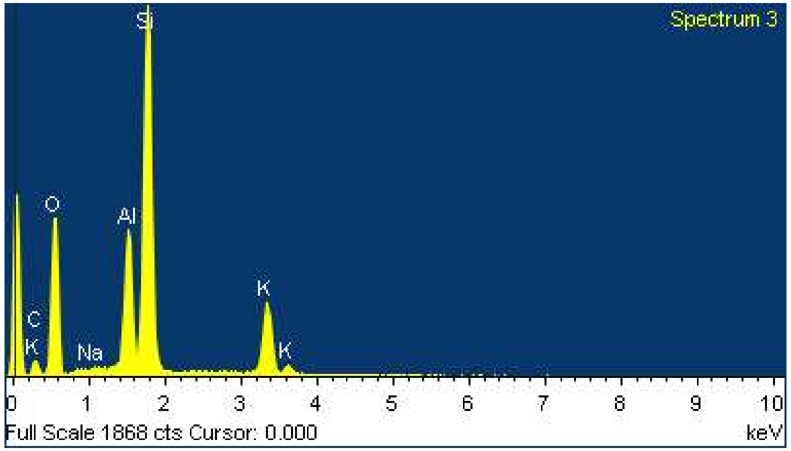

Element	Weight%	Atomic%
C K	9.70	15.90
O K	42.49	52.31
Na K	0.24	0.21
Al K	8.78	6.41
Si K	28.49	19.98
K K	10.29	5.18
Totals	100.00	

Mar 14 2017 MN UT16-MN-Jp1

14/03/2017 10:52:05

Sample: Jp1 1

Type: Default

ID:

Spectrum processing :

No peaks omitted

Processing option : All elements analyzed (Normalised)

Number of iterations = 5

Standard :

C CaCO3 1-Jun-1999 12:00 AM

O SiO2 1-Jun-1999 12:00 AM

Al Al2O3 1-Jun-1999 12:00 AM

Si SiO2 1-Jun-1999 12:00 AM

Ca Wollastonite 1-Jun-1999 12:00 AM

As InAs 1-Jun-1999 12:00 AM

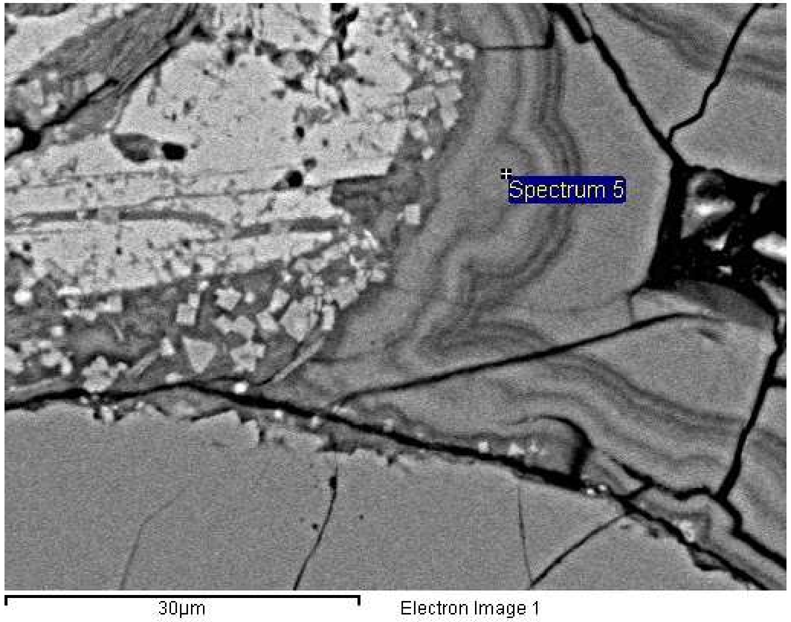

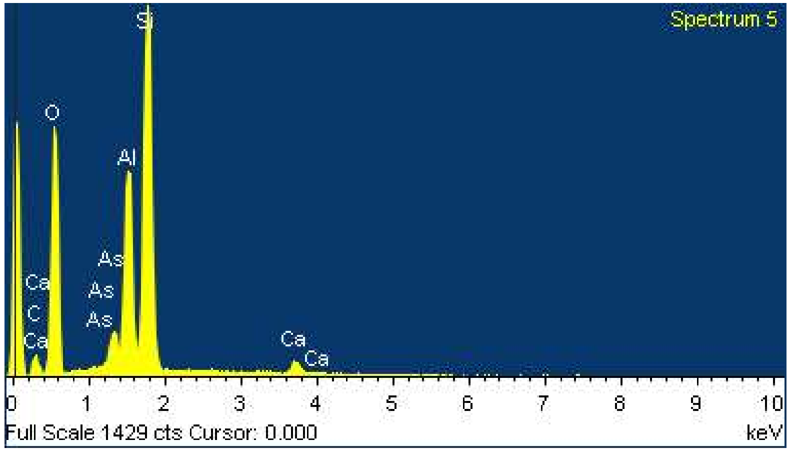

Element	Weight%	Atomic%
C K	11.92	18.78
O K	45.60	53.95
Al K	11.61	8.14
Si K	26.12	17.60
Ca K	1.47	0.69
As L	3.29	0.83
Totals	100.00	

Mar 14 2017 MN UT16-MN-Jp1

14/03/2017 11:28:56

Sample: Jp1 1

Type: Default

ID:

Spectrum processing :

No peaks omitted

Processing option : All elements analyzed (Normalised)

Number of iterations = 4

Standard :

C CaCO3 1-Jun-1999 12:00 AM

O SiO2 1-Jun-1999 12:00 AM

Al Al2O3 1-Jun-1999 12:00 AM

Si SiO2 1-Jun-1999 12:00 AM

Ca Wollastonite 1-Jun-1999 12:00 AM

As InAs 1-Jun-1999 12:00 AM

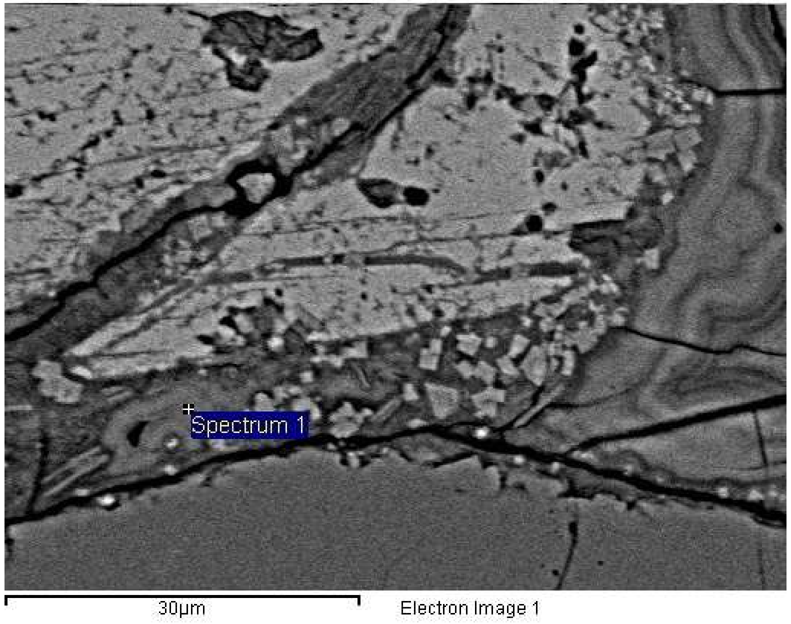

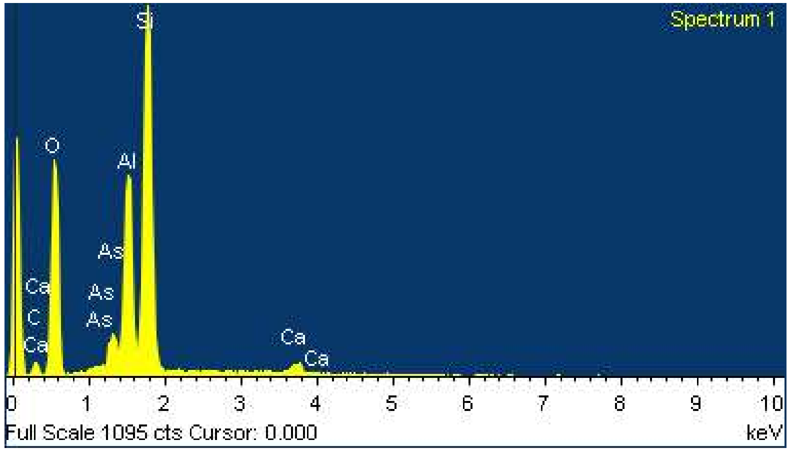

Element	Weight%	Atomic%
C K	8.53	13.92
O K	45.05	55.20
Al K	12.61	9.16
Si K	28.76	20.07
Ca K	1.43	0.70
As L	3.61	0.94
Totals	100.00	

Mar 14 2017 MN UT16-MN-Jp1

14/03/2017 13:21:12

Sample: Jp1 5

Type: Default

ID:

Spectrum processing :

No peaks omitted

Processing option : All elements analyzed (Normalised)

Number of iterations = 4

Standard :

C CaCO3 1-Jun-1999 12:00 AM

O SiO2 1-Jun-1999 12:00 AM

Si SiO2 1-Jun-1999 12:00 AM

Fe Fe 1-Jun-1999 12:00 AM

Element	Weight%	Atomic%

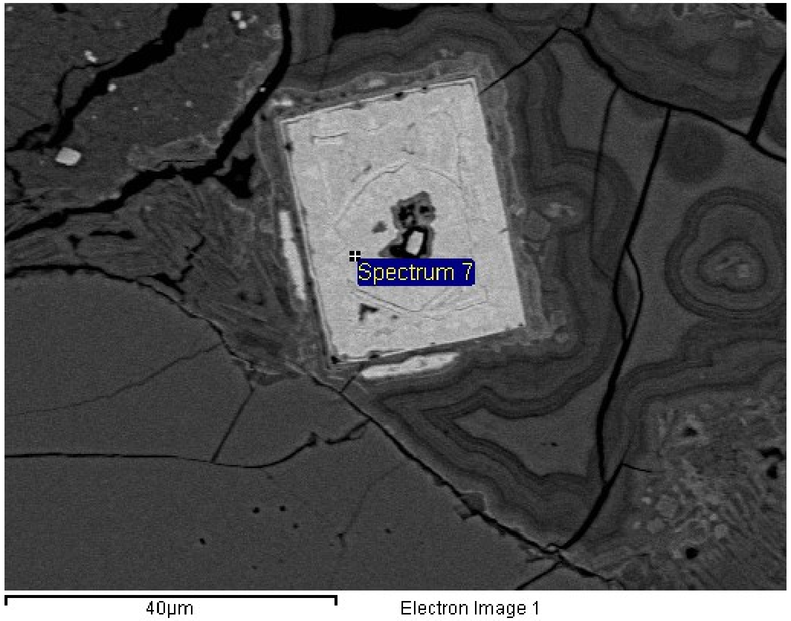

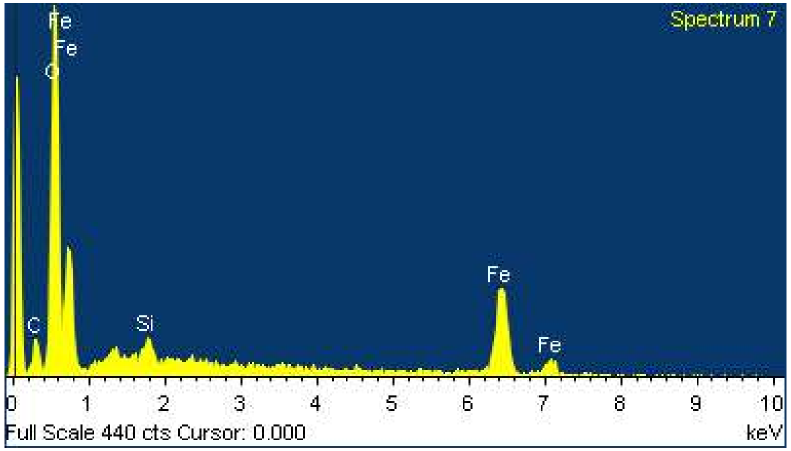

C K	8.99	16.72
O K	46.33	64.69
Si K	1.80	1.43
Fe K	42.88	17.15
Totals	100.00	

April 7 MN UT16-MN-Jp2

Sample: UT16-MN-Jp2 1

Type: Default

ID:

Spectrum processing :

No peaks omitted

Processing option : All elements analyzed (Normalised)

Number of iterations = 6

Standard :

C CaCO3 1-Jun-1999 12:00 AM

O SiO2 1-Jun-1999 12:00 AM

Al Al2O3 1-Jun-1999 12:00 AM

Si SiO2 1-Jun-1999 12:00 AM

Cl KCl 1-Jun-1999 12:00 AM

Element	Weight%	Atomic%

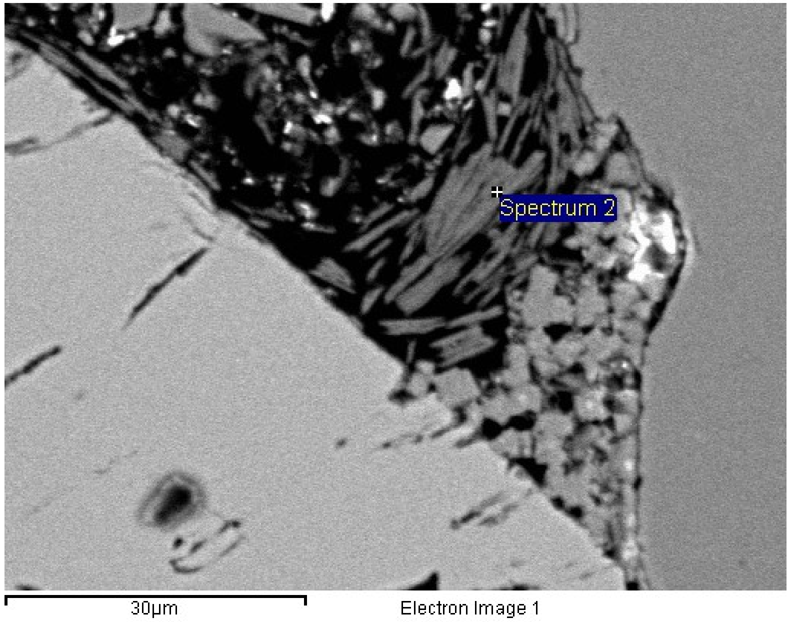

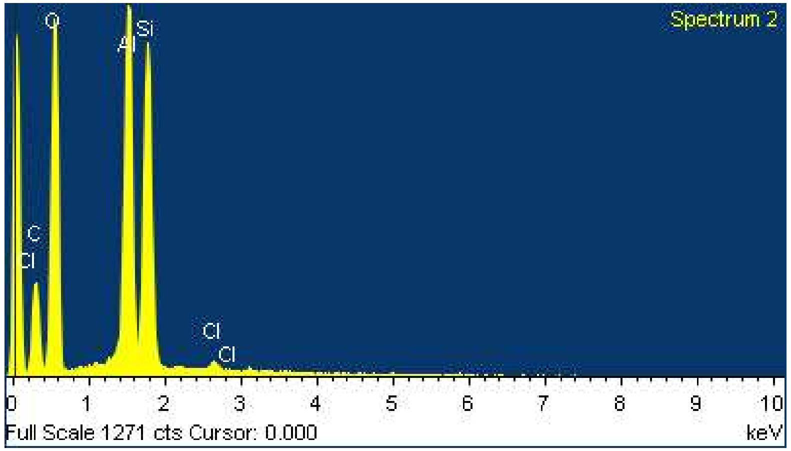

April 7 MN UT16-MN-Jp2

07/04/2017 12:42:44

Sample: UT16-MN-Jp2 1

Type: Default

ID:

Spectrum processing :

No peaks omitted

Processing option : All elements analyzed (Normalised)

Number of iterations = 6

Standard :

C CaCO3 1-Jun-1999 12:00 AM

O SiO2 1-Jun-1999 12:00 AM

Al Al2O3 1-Jun-1999 12:00 AM

Si SiO2 1-Jun-1999 12:00 AM

Cl KCl 1-Jun-1999 12:00 AM

Element	Weight%	Atomic%

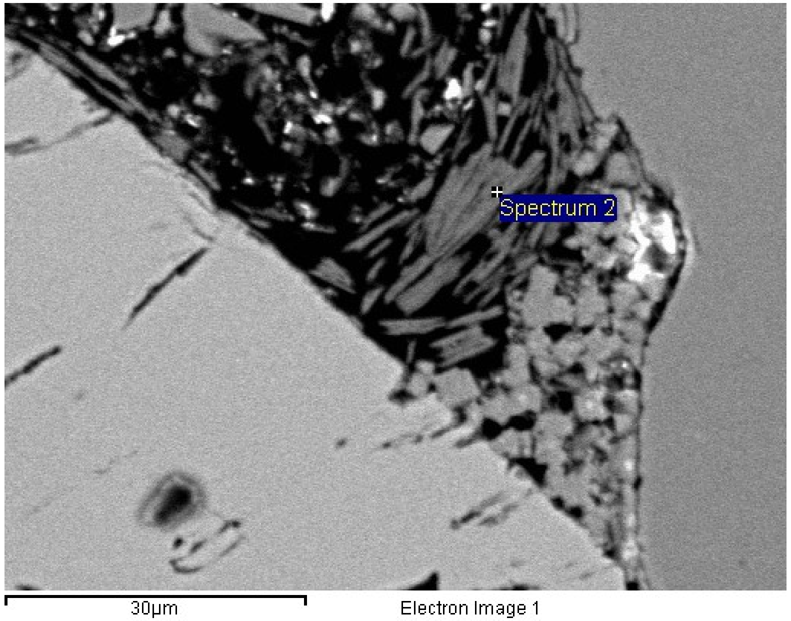

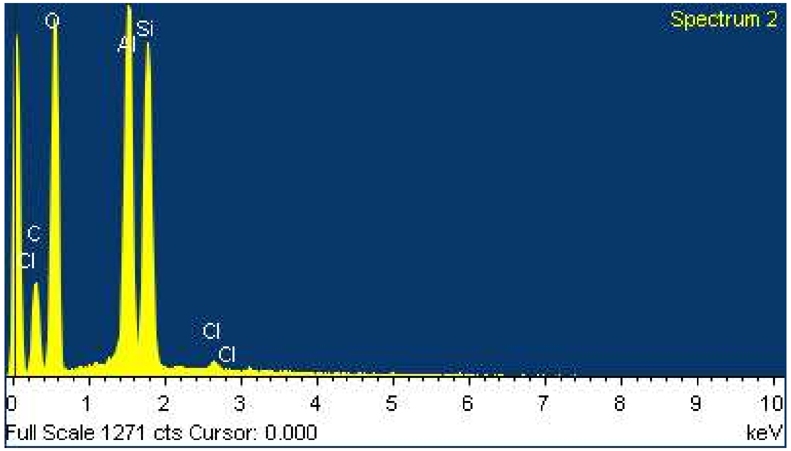

C K	25.75	35.42
O K	46.50	48.01
Al K	12.75	7.81
Si K	14.51	8.53
Cl K	0.50	0.23
Totals	100.00	

Too many elements to fit on page (3 max.) Please consider using the copy to clipboard function.

April 7 MN UT16-MN-Jp2

07/04/2017 13:08:18

Sample: UT16-MN-Jp2 1

Type: Default

ID:

Spectrum processing :

No peaks omitted

Processing option : All elements analyzed (Normalised)

Number of iterations = 5

Standard :

C   CaCO3   1-Jun-1999 12:00 AM

O   SiO2   1-Jun-1999 12:00 AM

Al   Al2O3   1-Jun-1999 12:00 AM

Si   SiO2   1-Jun-1999 12:00 AM

S   FeS2   1-Jun-1999 12:00 AM

K   MAD-10 Feldspar   1-Jun-1999 12:00 AM

Fe   Fe   1-Jun-1999 12:00 AM

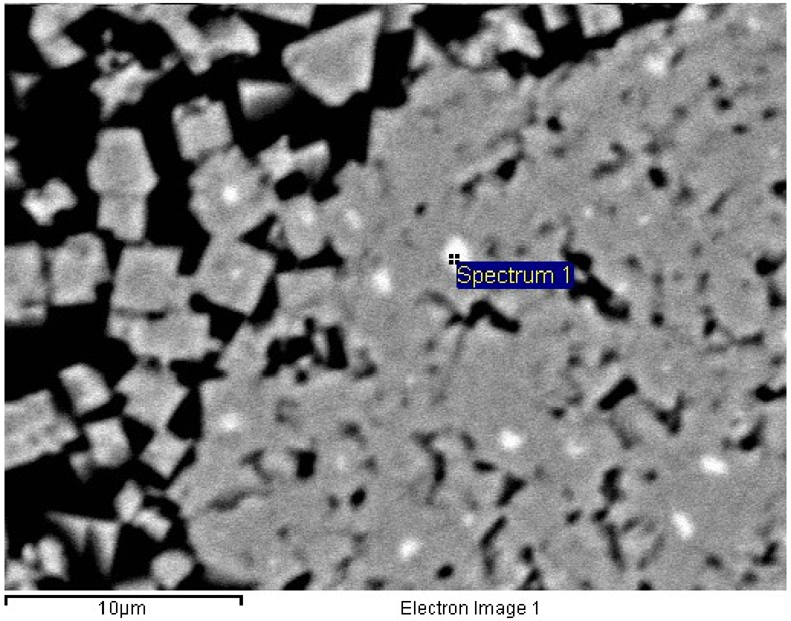

Electron Image 1

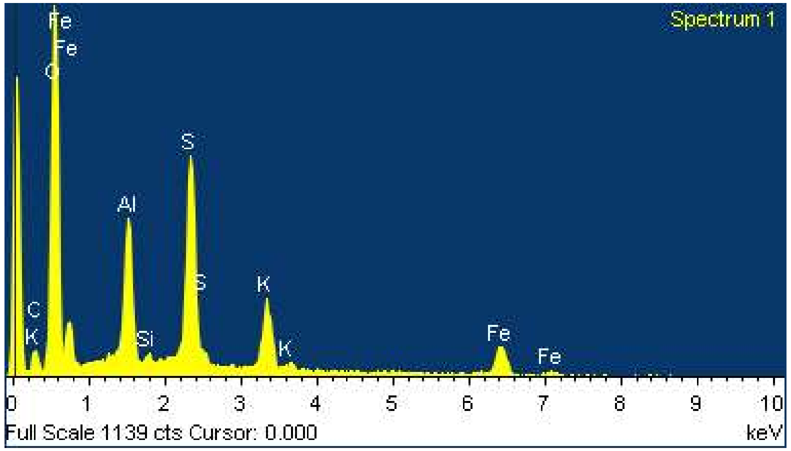

Element	Weight%	Atomic%
C K	9.16	14.83
O K	53.54	65.09
Al K	6.56	4.73
Si K	0.58	0.40
S K	13.67	8.29
K K	6.12	3.04
Fe K	10.38	3.62
Totals	100.00	

April 7 MN UT16-MN-Jp2

07/04/2017 13:09:03

Sample: UT16-MN-Jp2 1

Type: Default

ID:

Spectrum processing :

Peaks possibly omitted : 1.015, 6.375 keV

Processing option : All elements analyzed (Normalised)

Number of iterations = 5

Standard :

C CaCO3 1-Jun-1999 12:00 AM

O SiO2 1-Jun-1999 12:00 AM

Al Al2O3 1-Jun-1999 12:00 AM

S FeS2 1-Jun-1999 12:00 AM

K MAD-10 Feldspar 1-Jun-1999 12:00 AM

Element	Weight%	Atomic%

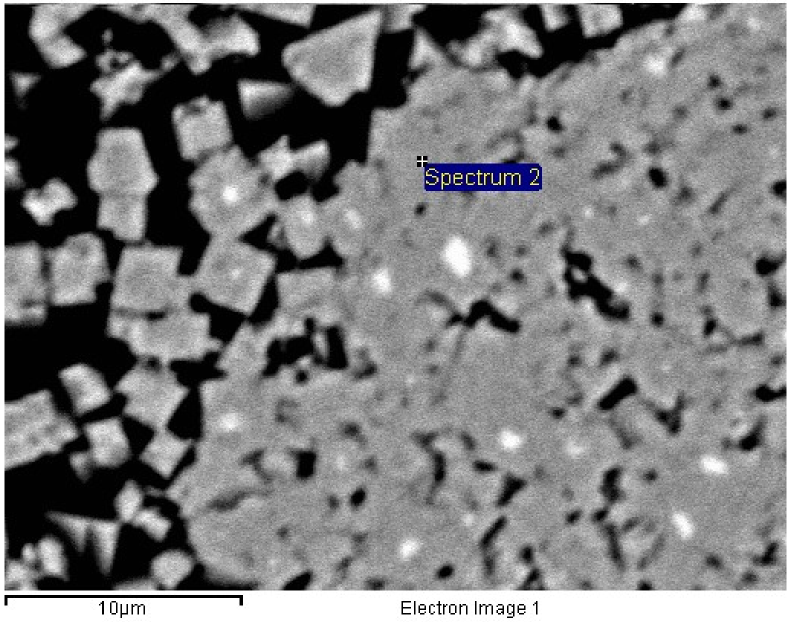

Electron Image 1

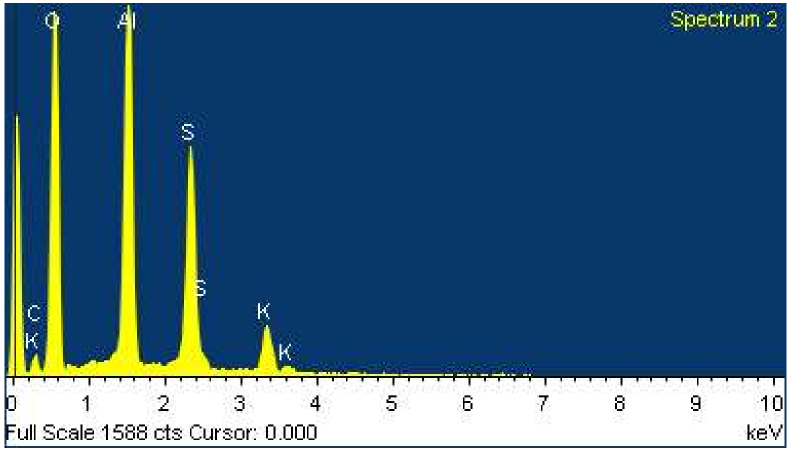

C K	8.32	12.93
O K	55.03	64.26
Al K	17.35	12.01
S K	14.93	8.70
K K	4.38	2.09
Totals	100.00	
